# Development of a Rapid *in vivo* Assay to Evaluate the Efficacy of IRE1-specific Inhibitors of the Unfolded Protein Response Using Medaka Fish

**DOI:** 10.1247/csf.19032

**Published:** 2019-12-26

**Authors:** Byungseok Jin, Tokiro Ishikawa, Mai Taniguchi, Satoshi Ninagawa, Tetsuya Okada, Shigehide Kagaya, Kazutoshi Mori

**Affiliations:** 1 Department of Biophysics, Graduate School of Science, Kyoto University, Kyoto 606-8502, Japan; 2 Nippon Kayaku Co., Ltd.

**Keywords:** endoplasmic reticulum, inhibitor screening, mRNA splicing, phenotypic assay, unfolded protein response

## Abstract

Three types of transmembrane protein, IRE1α/IRE1β, PERK, and ATF6α/ATF6β, are expressed ubiquitously in vertebrates as transducers of the unfolded protein response (UPR), which maintains the homeostasis of the endoplasmic reticulum. IRE1 is highly conserved from yeast to mammals, and transmits a signal by a unique mechanism, namely splicing of mRNA encoding XBP1, the transcription factor downstream of IRE1 in metazoans. IRE1 contains a ribonuclease domain in its cytoplasmic region which initiates splicing reaction by direct cleavage of *XBP1* mRNA at the two stem loop structures. As the UPR is considered to be involved in the development and progression of various diseases, as well as in the survival and growth of tumor cells, UPR inhibitors have been sought. To date, IRE1 inhibitors have been screened using cell-based reporter assays and fluorescent-based *in vitro* cleavage assays. Here, we used medaka fish to develop an *in vivo* assay for IRE1α inhibitors. IRE1α, IRE1β, ATF6α and ATF6β are ubiquitously expressed in medaka. We found that IRE1α/ATF6α-double knockout is lethal, similarly to IRE1α/IRE1β- and ATF6α/ATF6β-double knockout. Therefore, IRE1 inhibitors are expected to confer lethality to ATF6α-knockout medaka but not to wild-type medaka. One compound named K114 was obtained from 1,280 compounds using this phenotypic screening. K114 inhibited ER stress-induced splicing of *XBP1* mRNA as well as reporter luciferase expression in HCT116 cells derived from human colorectal carcinoma, and inhibited ribonuclease activity of human IRE1α *in vitro*. Thus, this phenotypic assay can be used as a quick test for the efficacy of IRE1α inhibitors *in vivo*.

## Introduction

Newly synthesized secretory and transmembrane proteins are folded and assembled in the endoplasmic reticulum (ER) with assistance from ER-localized molecular chaperones and folding enzymes, which are abundantly and ubiquitously expressed. Because these proteins play critical roles in intercellular communication, maintenance of their quality is essential for numerous phenomena. For example, insulin binds to its receptor to regulate blood glucose levels only when they both have the correct three dimensional structures (Bukau *et al.*, 2006).

The unfolded protein response (UPR) is activated by so-called ER stress, the accumulation of unfolded or misfolded proteins in the ER. The UPR consists of translational and transcriptional programs mediated by three types of ubiquitously expressed transmembrane proteins in the ER, namely IRE1, PERK, and ATF6. Activation of PERK attenuates translation generally and transiently to decrease the burden on the ER. Activation of ATF6 transcriptionally induces ER-localized molecular chaperones and folding enzymes to augment folding capacity in the ER. Activation of IRE1 together with activation of ATF6 transcriptionally induces components of ER-associated degradation to enhance retrotranslocation of misfolded proteins from the ER to the cytosol for ubiquitin-dependent proteasomal degradation. These act together to ensure maintenance of the homeostasis of the ER (Mori, 2000; Walter and Ron, 2011; Yamamoto *et al.*, 2007).

Failure of such maintenance leads to the development and progression of various diseases (Wang and Kaufman, 2012). For example, PERK-knockout (KO) mice develop diabetes mellitus (Harding *et al.*, 2001), while mutation in PERK causes Wolcott-Rallison syndrome in humans, which is characterized by neonatal/early-onset diabetes (Delepine *et al.*, 2000). On the other hand, tumor cells survive under hypoxic and low-nutrient conditions and grow in nude mice by activating the IRE1 (Romero-Ramirez *et al.*, 2004) and PERK (Bi *et al.*, 2005) pathways. Prognosis of patients with triple negative breast cancer is poor when the IRE1 pathway is activated (Chen *et al.*, 2014). Accordingly, inhibitors of these transmembrane proteins are of potential use as anti-cancer drugs (Urra *et al.*, 2016).

IRE1 is a type I transmembrane protein with protein kinase and ribonuclease domains facing the cytoplasm. When activated by ER stress-induced oligomerization and autophosphorylation, IRE1 transmits a signal by a unique mechanism, namely unconventional (frame switch) splicing of *HAC1* mRNA in yeast and *XBP1* mRNA in metazoans. This removes an intron of 252 and 26 nucleotides, respectively, which causes frame shift at the splice site, resulting in the replacement of their C-terminal amino acids (Mori, 2003). This splicing is initiated by direct cleavage of their mRNAs by activated IRE1 (Sidrauski and Walter, 1997). Spliced *HAC1* and *XBP1* mRNAs are translated to produce active forms of the transcription factors HAC1 and XBP1, respectively, culminating in transcriptional induction of their target genes in the nucleus (Mori, 2009).

To date, IRE1 inhibitors have been sought basically using two methods, namely cell culture-based reporter assays (Papandreou *et al.*, 2011; Ri *et al.*, 2012), in which firefly luciferase gene is fused to the second open reading frame present in unspliced *XBP1* mRNA such that firefly luciferase activity is expressed after ER stress-induced splicing (see Fig. 2E); and fluorescent-based *in vitro* FRET-derepression assay (Cross *et al.*, 2012; Volkmann *et al.*, 2011), in which a part of *XBP1* mRNA containing the splice site becomes fluorescent after direct cleavage by the purified cytoplasmic region of IRE1 (IRE1-C) *in vitro*.

We considered that it would be highly useful if we could establish an assay by which the efficacy of IRE1 inhibitors could be quickly assessed *in vivo*. Here, we attempted to develop such an assay using medaka fish, in which, similarly to mammals, all three UPR transducers—IRE1, PERK and ATF6—are functional.

## Materials and Methods

### Statistics

Statistical analysis was conducted using Student’s t-test, with probability expressed as *p<0.05, **p<0.01, and ***p<0.001 for all figures.

### Fish

Medaka southern strain cab was used as wild-type fish. Fish were maintained in a recirculating system with a 14:10 h light:dark cycle at 27.5°C. All experiments were performed in accordance with the guidelines and regulations established by the Animal Research Committee of Kyoto University (approval number: H2819). IRE1α and ATF6α knockout medaka have been described previously (Ishikawa *et al.*, 2017, 2013). Medaka embryos were harvested immediately after fertilization, incubated at 30°C, and observed by light optical microscopy.

### Genotyping of IRE1α and ATF6α

Embryos were individually suspended in 30 μl of lysis buffer (10 mM NaOH and 0.2 mM EDTA), boiled for 10 min, and neutralized by the addition of 30 μl of 40 mM Tris/HCl, pH 8.0. Amplified PCR fragments were digested by restriction enzymes Hpy1881 and Xsp1 for IRE1α and ATF6α, respectively, and subjected to agarose gel electrophoresis.

### Cell culture

HCT116 cells (ATCC CCL-247) were cultured in Dulbecco’s modified Eagle’s medium (glucose 4.5 g/liter) supplemented with 10% fetal bovine serum, 2 mM glutamine and antibiotics (100 U/ml penicillin and 100 μg/ml streptomycin) at 37°C in a humidified 5% CO_2_/95% air atmosphere. T47D cells (ATCC HTB-133) were cultured in RPMI1640 medium with 10% fetal bovine serum and antibiotics (100 U/ml penicillin and 100 μg/ml streptomycin). Medium pH was adjusted to 6.0 by the addition of 5% MES buffer.

### RT-PCR-mediated detection of *XBP1* mRNA splicing

Total RNA was extracted from embryos, HCT116 cells, and T47D cells by the guanidine phenol/chloroform method using ISOGEN (Nippon Gene). Whole RNA from embryos and 3 μg RNA from cells were treated with M-MLV reverse transcriptase (Invitrogen), amplified with Ex-Taq polymerase (Takara) using XBP1-specific primers, and subjected to agarose gel electrophoresis.

### Construction of a stable cell line expressing XBP1-luciferase

pDEST26 vector (Invitrogen) carrying the XBP1-firefly luciferase gene under control of the CMV promoter and pRL-SV40P vector (Addgene) carrying the renilla luciferase gene (1 μg each) were co-transfected into HCT116 cells using PEI MAX transfection reagent. Strain #11 cells showed stable expression of firefly and renilla luciferase activities among 23 colonies selected by neomycin resistance. Transient transfection of pDEST26 vector carrying the XBP1-firefly luciferase gene (3 μg) and pRL-SV40P vector carrying the renilla luciferase gene (0.2 μg) was also carried out using PEI MAX transfection reagent.

### Luciferase assay

HCT116 cells stably expressing XBP1-luciferase were seeded at 3.5×10^4^ cells/well in a 96-well plate and treated with DMSO, 2 μg/ml tunicamycin, and 45 μM 4μ8C or 50 μM K114 for 18 hr. After washing with PBS, cells were lysed with Luciferase Assay Lysis Buffer, and luciferase assay was performed using PicaGene Dual-luciferase reporter assay reagent (Promega). Relative luciferase activity was defined as the ratio of firefly luciferase activity to renilla luciferase activity.

### Purification of IRE1-C using baculovirus expression system

pDEST10 vector (Invitrogen) carrying IRE1-C (500 ng) was transduced into *E coli* strain DH10Bac and transformed colonies were obtained by white and blue selection. The formation of bacmid was confirmed by colony PCR. SF9 insect cells (Invitrogen) were transfected with 8 μg of the resulting Bacmid purified using Mini-prep (Qiagen) and cultured in 50 ml of Grace’s Insect Medium (Gibco) with 10% fetal bovine serum for 4 days, allowing virus amplification. Collected virus was amplified again in SF9 cells in 500 ml medium for 3 days. The cells were harvested, lysed in 30 ml lysis buffer (50 mM Tris/HCl, pH 8.0, containing 150 mM NaCl, 10 mM imidazole, 5 mM 2-mercaptoethanol, protease inhibitor cocktail and 0.1% Triton X-100) on ice for 30 min, sonicated, and then centrifuged at 16,000 rpm for 1 h. IRE1-C was purified from supernatant using nickel column (cOmplete His-Tag Purification Resin; Roche). The protein enrichment step was performed using Amicon-Ultra-15 (Merck Millipore) and buffer was changed to lysis:glycerol (9:1) buffer using a PD-10 column (GE Healthcare).

### *in vitro* transcription of a part of *XBP1* mRNA

pDEST26 vector carrying the XBP1-firefly luciferase gene was cut by EcoRI and purified using phenol/chloroform. 100 ng/μl DNA were reacted with T7 polymerase for 1.5 h in T7 Transcription buffer (0.2 M Tris/HCl, pH 8.0, containing 40 mM MgCl_2_, 10 mM spermidine-(HCl)_3_, and 125 mM NaCl), which in addition included 0.1 M dithiothreitol, RNase inhibitor (RNase OUT; Invitrogen) and 3.3 mM each of ATP, CTP, GTP and UTP (or ^32^P-UTP).

### *in vitro* assay for IRE1 ribonuclease activity

We used the published procedure (Sidrauski and Walter, 1997) as reference. IRE1-C (30 ng) was incubated with *in vitro* transcribed *XBP1* mRNA (approximately 5 ng and 50 ng of ^32^P-labeled and unlabeled mRNA, respectively) in kinase buffer (20 mM HEPES. pH 7.6, containing 1 mM dithiothreitol, 10 mM Mg(OAc)_2_, 50 mM KOAc and RNase OUT) at 37°C for 30 min and then cooled down on ice. An equal volume of loading buffer (18 mM EDTA, 0.025% SDS, 0.025% xylene cyanol, 0.025% bromophenol blue, and 95% formamide) was added and samples were denatured at 90°C for 5 min. Samples were then separated using Denaturing Urea-PAGEs (Novex TBE Urea gel, 6%) which was run for 1.5 h, and autoradiographed or stained with SYBR Gold Nucleic acid gel stain agent (Invitrogen) for 30 min.

### Quantitative RT-PCR

Quantitative RT-PCR analysis was carried out as described previously (Ishikawa *et al.*, 2013) using the SYBR Green method (Applied Biosystems) and a pair of primers, namely 5'-TCTCAGATCTTTTCTACAGCTTCTGA-3' and 5'-TGTCTTTTGTCAGGGGTCTTTCA-3' for human BiP, 5'-TAAGAGCCCGGATGCTGAAG-3' and 5'-TCGTCTATTAGCATCTGAGAGTGT-3' for human ERdj4, 5'-ACCTATGTTTCACCTCCTGGA-3' and 5'-CAGTCAGCCAAGCCAGAGAA-3' for human CHOP, 5'-CTGCTGAGTCCGCATCAGGT-3' and 5'-GAGTCAATACCGCCAGAATCCA-3' for human XBP1(S), 5'-AGTTCCTCCTGACACCAATA-3' and 5'-GTCGACTCAGAATCCCAAAT-3' for human EDEM1, and 5'-GACCCCTTCATTGACCTCAA-3' and 5'-TTGACGGTGCCATGGAATT-3' for human GAPDH.

### Cell growth assay

HCT116 and T47D cells were seeded at 2,000 cells per well in a 96-well plate and treated with various concentrations of 4μ8C or K114 from the next day for 3 days. The number of viable cells was then estimated using The CellTiter-Glo Luminescent Cell Viability Assay agent (Promega), in which luminescent output correlates with cell number.

## Results and Discussion

Both IRE1 and ATF6 consist of two related forms, IRE1α/IRE1β and ATF6α/ATF6β, respectively, in vertebrates. Medaka IRE1α, medaka IRE1β, and mouse IRE1α are ubiquitously expressed (Ishikawa *et al.*, 2011; Urano *et al.*, 2000), whereas mouse IRE1β is expressed only in the gut (Bertolotti *et al.*, 2001). ATF6α and ATF6β are ubiquitously expressed in both medaka and mice (Ishikawa *et al.*, 2011; Yamamoto *et al.*, 2007). We previously showed that IRE1α-KO, IRE1β-KO, ATF6α-KO and ATF6β-KO medaka show no obvious phenotype but that both IRE1α/β-double KO (DKO) and ATF6α/β-DKO cause lethality in medaka (Ishikawa *et al.*, 2017, 2013).

**Fig. 1:**
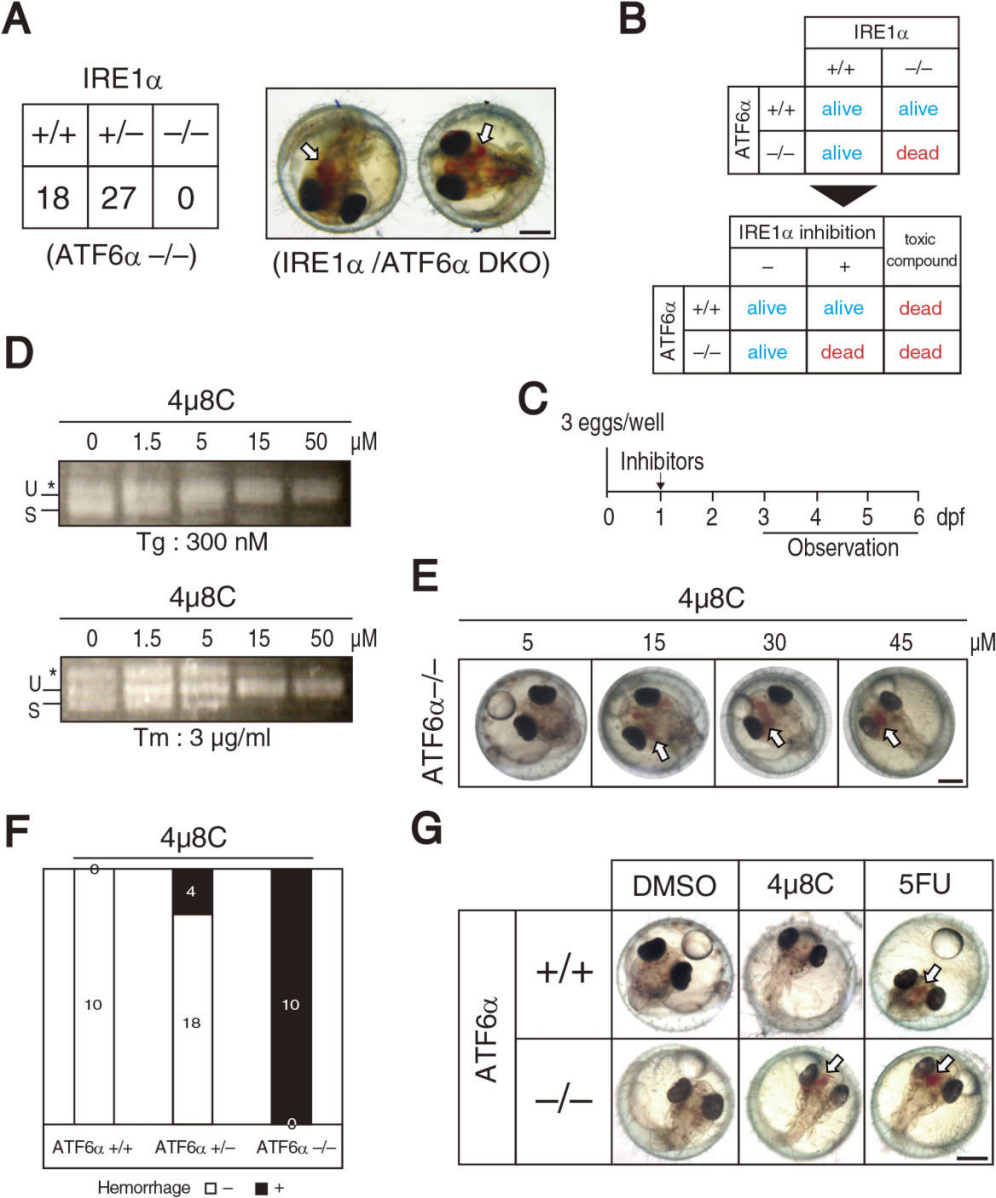
Strategy for obtaining IRE1α inhibitors. (A) Male IRE1α+/– medaka and female ATF6α–/– medaka were crossed, and the resulting 45 hatched fish were genotyped (left). Embryos exhibiting intracerebral hemorrhage at 4 dpf were genotyped to be IRE1α/ATF6α-DKO (right). Scale bar: 250 μm. (B) Expected phenotypes of ATF6α+/+ and ATF6α–/– medaka treated with IRE1 inhibitors or toxic compounds. (C) Procedure for screening. Three fertilized eggs were placed into each well of a 96-well plate, treated, and observed as indicated. (D) ATF6α+/+ embryos at 1 dpf were treated with 300 nM thapsigargin (Tg) or 3 μg/ml tunicamycin (Tm) together with the indicated concentration of 4μ8C for 24 h. Total RNA were prepared and subjected to RT-PCR. U and S indicate RT-PCR product corresponding to unspliced and spliced *XBP1* mRNA, respectively. The asterisk denotes a heteroduplex of unspliced and spliced *XBP1* mRNA. (E) ATF6α–/– embryos at 1 dpf were treated with the indicated concentrations of 4μ8C and photographed at 6 dpf. Scale bar: 250 μm. (F) Intracerebral hemorrhage ratio of embryos with the indicated genotypes after treatment with 4μ8C (30 μM). (G) ATF6α+/+ and ATF6α–/– embryos at 1 dpf were treated with DMSO, 4μ8C (30 μM), and 5FU (0.3 mg/ml), and photographed at 6 dpf. Scale bar: 250 μm.

The present study shows that IRE1α/ATF6α-DKO also causes embryonic lethality and that IRE1α/ATF6α-DKO embryos exhibit intracerebral hemorrhage before hatching [medaka hatch at 7 day post-fertilization (dpf)] (Fig. 1A). Because intracerebral hemorrhage is induced in zebrafish by a variety of gene mutations and morpholino-mediated gene knockdown, as well as after treatment with various compounds (Eisa-Beygi and Rezaei, 2016), we were unable to determine the cause of intracerebral hemorrhage at this stage. Instead, based on this lethal phenotype we came up with the strategy of developing *in vivo* assay for IRE1α inhibitors. Namely, IRE1α inhibitors would confer a lethal phenotype to ATF6α–/– medaka but not to wild-type (ATF6α+/+) medaka. Any toxic compound can confer a lethal phenotype to ATF6α–/– medaka but would affect ATF6α+/+ medaka as well (Fig. 1B). Thus, IRE1α-specific inhibitors would be expected to induce intracerebral hemorrhage before hatching only in ATF6α–/– medaka grown in 96-well plates (Fig. 1C). Indeed, 4μ8C, a specific inhibitor of IRE1α ribonuclease (Cross *et al.*, 2012), inhibited splicing of *XBP1* mRNA in embryos treated with the ER stress inducers thapsigargin or tunicamycin (Fig. 1D) and induced intracerebral hemorrhage at 4 dpf in ATF6α–/– embryos in a dose- and genotype-dependent manner (Fig. 1E and1F). In contrast, 5-fluorouracil (5FU), a potent anti-cancer reagent, induced intracerebral hemorrhage at 6 dpf in both ATF6α+/+ and ATF6α–/– medaka (Fig. 1G) due to its toxicity, as expected.

**Fig. 2:**
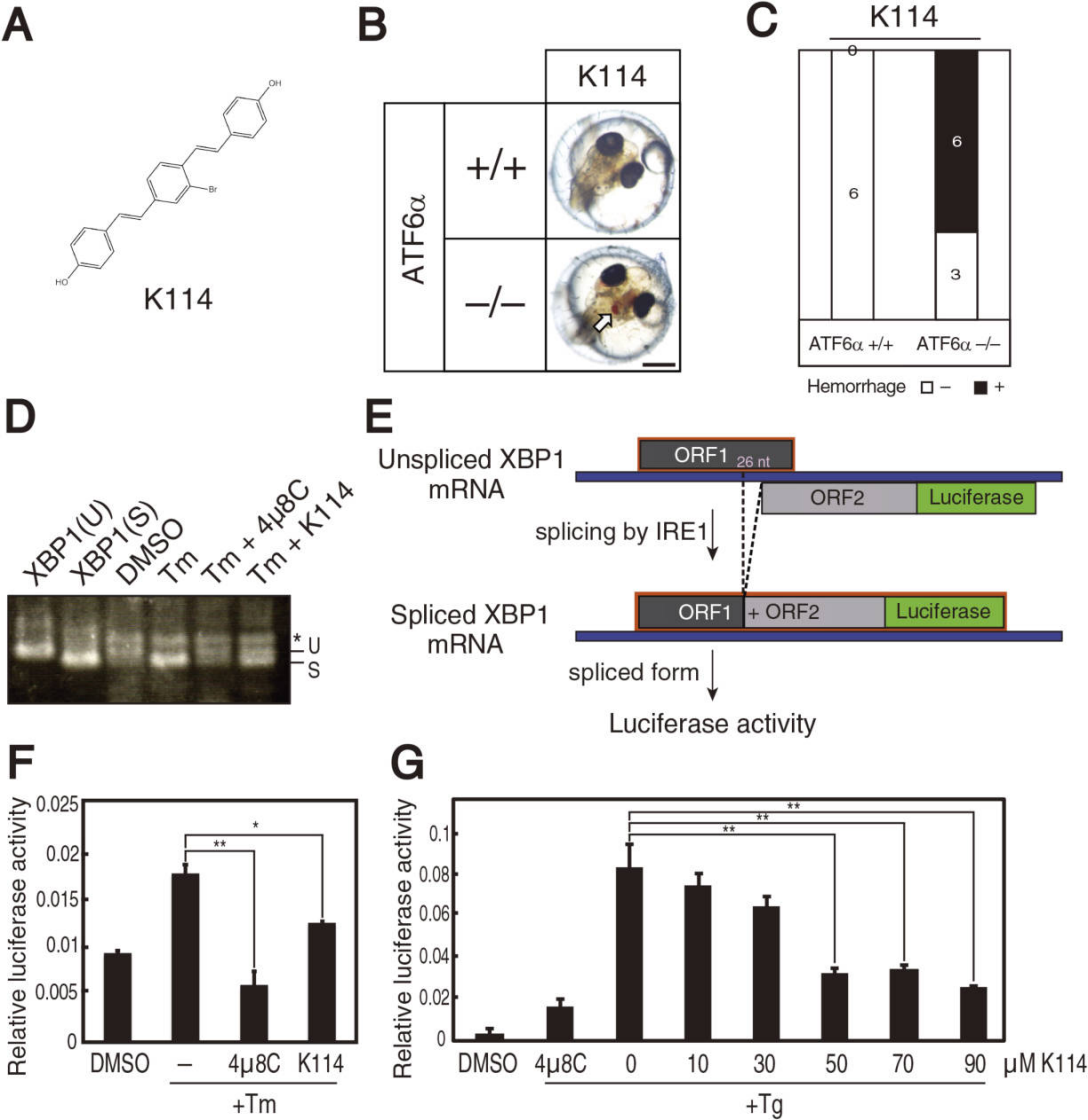
Effect of K114 on splicing of *XBP1* mRNA. (A) Chemical structure of K114. (B) ATF6α+/+ and ATF6α–/– embryos at 1 dpf were treated with 30 μM K114 for 5 days, and then photographed. (C) Intracerebral hemorrhage ratio of embryos with the indicated genotypes after treatment with K114 (30 μM). (D) HCT116 cells were treated with 2 μg/ml tunicamycin with or without 4μ8C (45 μM) or K114 (50 μM) for 18 h. Total RNA were prepared and subjected to RT-PCR, as in Fig. 1D. (E) Schematic representation of the XBP1-luciderase reporter system. (F) HCT116 cells stably expressing XBP1-firefly luciferase reporter were treated with 2 μg/ml tunicamycin with or without 4μ8C (45 μM) or K114 (50 μM) for 18 h, and then cellular luciferase activity was determined. (G) HCT116 cells transiently expressing XBP1-firefly luciferase reporter were treated with 300 nM thapsigargin together with the indicated concentrations of K114 or 4μ8C (45 μM) for 18 h, and then cellular luciferase activity was determined.

We screened LOPAC1280 (Merck), a collection of 1,280 pharmacologically active compounds, at 30 μM, and discovered one compound, designated K114 (Fig. 2A), as a candidate IRE1α inhibitor, on the basis that it induced intracerebral hemorrhage in ATF6α–/– embryos but not in ATF6α+/+ embryos (Fig. 2B and 2C). Importantly, K114 inhibited splicing of *XBP1* mRNA in the HCT116 diploid cell line derived from human colorectal carcinoma (Roschke *et al.*, 2002) treated with tunicamycin (Fig. 2D). Due to the lower quantitativity of RT-PCR analysis, we employed a reporter assay using the XBP1-firefly luciferase construct, which produces luciferase activity after IRE1-mediated splicing of *XBP1* mRNA (Fig. 2E). K114 inhibited tunicamycin-triggered induction of luciferase activity in HCT116 cells stably expressing XBP1-firefly luciferase reporter, albeit weakly compared with 4μ8C (Fig. 2F). Transient expression system of XBP1-firefly luciferase reporter in HCT116 cells revealed that the IC50 of K114 was 35.9 μM (Fig. 2G).

**Fig. 3:**
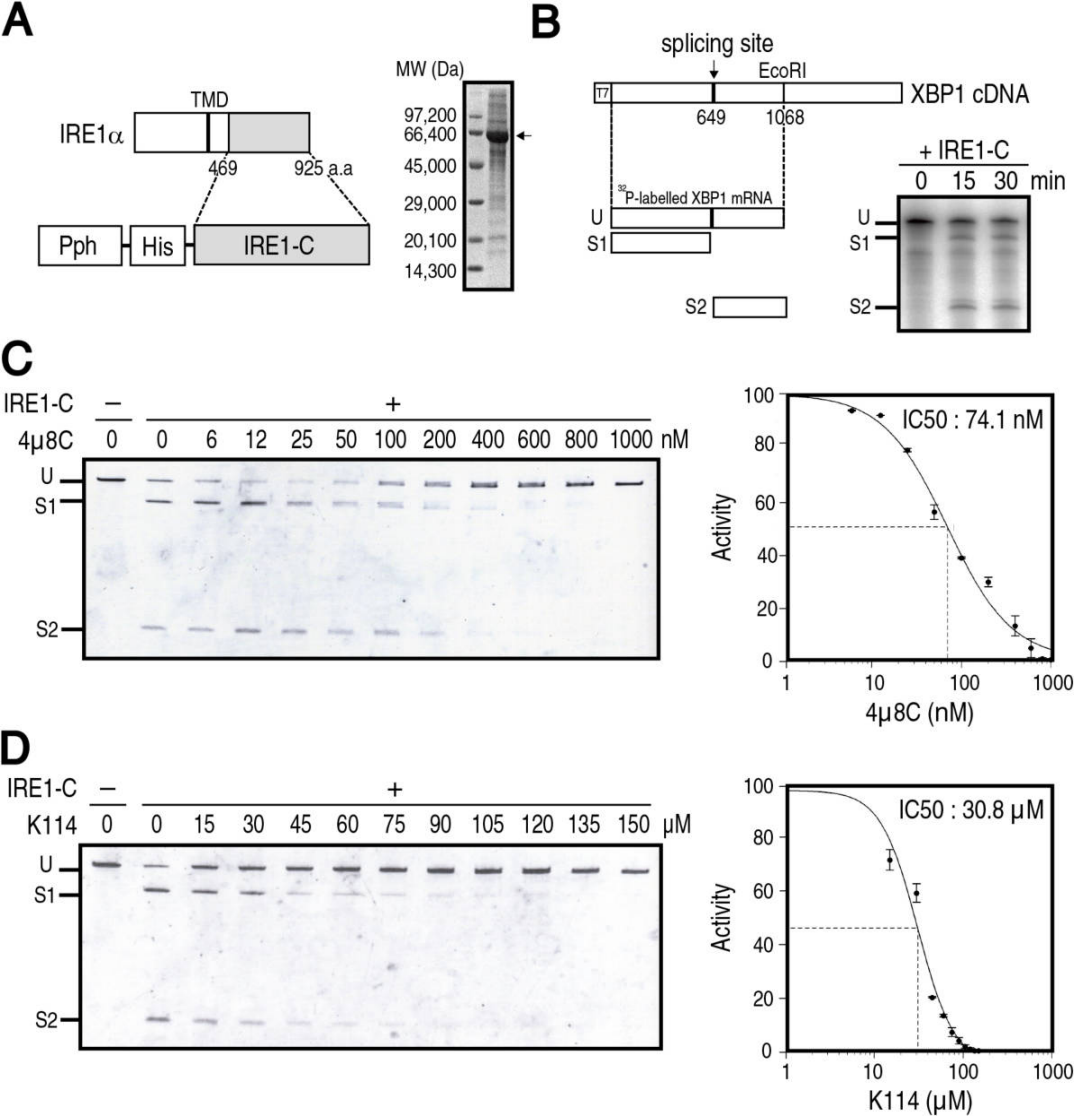
Effect of K114 on IRE1α ribonuclease activity *in vitro*. (A) Schematic representation of the strategy to express IRE1-C using a baculovirus system (left) and SDS-PAGE (10% gel) analysis of IRE1-C purified from SF9 insect cells (right, arrow). (B) Schematic representation of a part of *XBP1* mRNA as a substrate of IRE1-C (left). Unspliced fragment (U) was cleaved to produce spliced fragments (S1 and S2). ^32^P-labeled *XBP1* mRNA was incubated with purified IRE1-C for the indicated periods, separated by polyacrylamide gel, and autoradiographed (right). (C) (D) Unlabeled *XBP1* mRNA was incubated with (+) or without (–) purified IRE1-C for 30 min in the presence of the indicated concentration of 4μ8C (C) or K114 (D), separated by urea-containing polyacrylamide gel, and stained with SyberGold (n=3). Intensity of each band was determined. Summation of S1 and S2 divided by summation of U, S1 and S2 obtained without 4μ8C or K114 is taken as 100%. The IC50 was calculated by nonlinear regression using Quest Graph^TM^ IC50 Calculator (AAT Bioquest).

We next examined whether K114 inhibits the ribonuclease activity of IRE1α *in vitro*, similarly to 4μ8C. To this end, the His-tagged cytoplasmic region of IRE1α (IRE1-C) was expressed in insect cells under control of polyhedrin promoter (Pph), and was then purified from them using a nickel column. Purified IRE1-C exhibited a major band higher than the expected molecular weight of 52 kDa (Fig 3A), suggesting that IRE1-C might be hyper-phosphorylated via overexpression-based oligomerization, as shown previously (Rubio *et al.*, 2011; Wiseman *et al.*, 2010). IRE1-C cleaved at the expected sites on a part of *XBP1* mRNA transcribed *in vitro* with ^32^P-UTP (Fig. 3B), as reported previously (Cross *et al.*, 2012). Both 4μ8C and K114 inhibited *in vitro* cleavage of *XBP1* mRNA by purified IRE1-C in a dose-dependent manner (data not shown). To facilitate handling of this *in vitro* assay, we devised a non-radioisotopic method, in which we first incubated an approximately 10-fold greater amount of *in vitro* translated and unlabeled *XBP1* mRNA with IRE1-C, separated the cleaved fragments from uncleaved mRNA using urea-containing polyacrylamide gel, and then stained them with SyberGold. As a result, the assay became more quantitative; using it, we determined an IC50 for 4μ8C of 74.1 nM (Fig. 3C), which is similar to the IC50 of 61.6 nM reported previously (Cross *et al.*, 2012). An IC50 for K114 was determined to be 30.8 μM (Fig. 3D), which is comparable to the IC50 of 35.9 μM determined by the above reporter assay. It should be noted that the effective concentration of 4μ8C in medaka was higher by three orders of magnitude than that *in vitro* (see Fig. 1). This is because 4μ8C forms a Schiff base with lysine residues of IRE1, and is therefore easily inactivated nonspecifically *in vivo* prior to specific inhibition of IRE1 (Cross *et al.*, 2012).

**Fig. 4:**
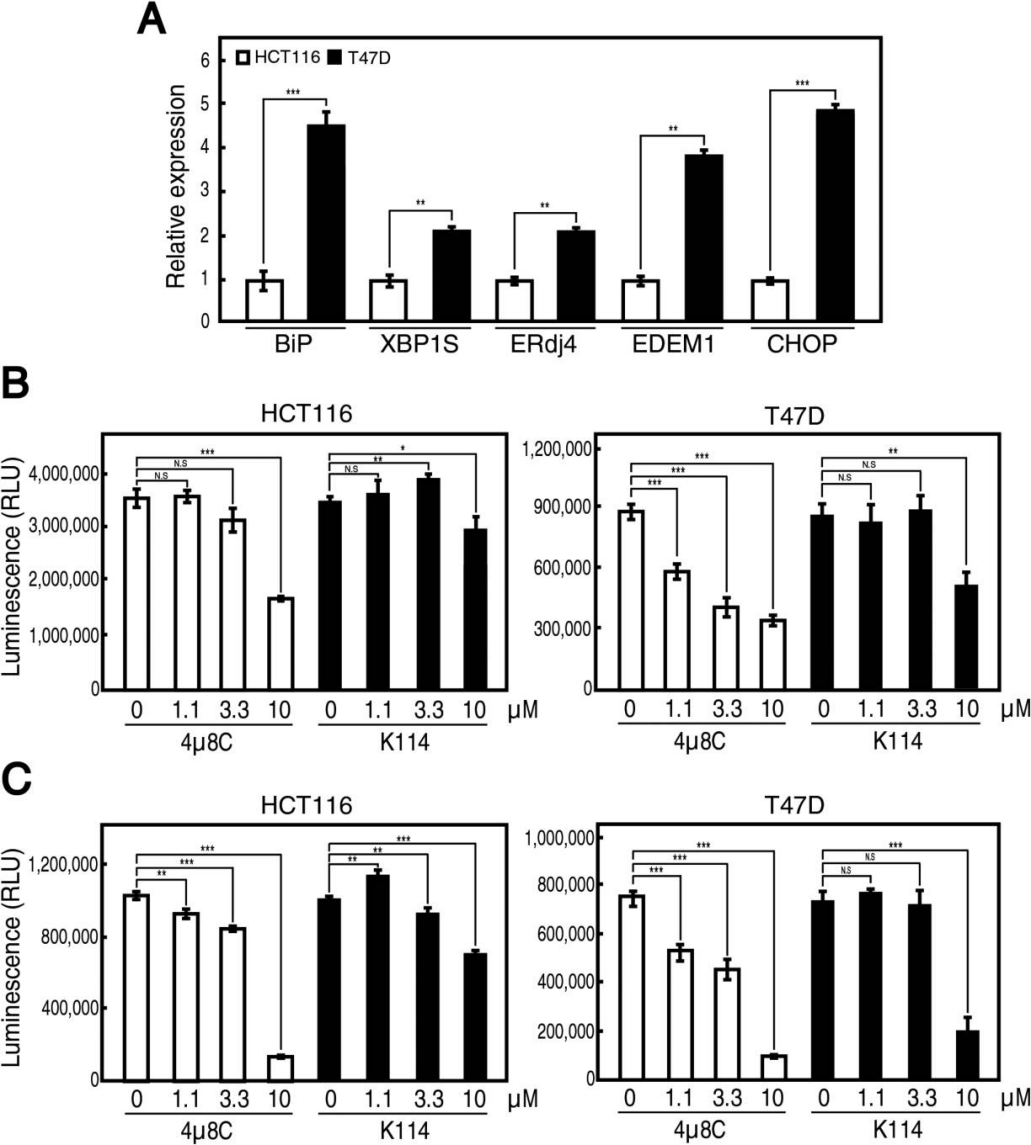
Effect of K114 on growth of HCT116 and T47D cells. (A) Total RNA prepared from unstressed HCT116 and T47D cells was subjected to quantitative RT-PCR. The expression level of indicated mRNA is normalized with the level of GAPDH mRNA (n=3). The level of each mRNA in T47D cells is then shown in comparison with that in HCT116 cells. (B) (C) HCT116 and T47D cells were treated with the indicated concentration of 4μ8C or K114 at pH 7.2 (B) or at pH 6.0 (C) for 3 days and subjected to cell growth assay. This experiment was conducted twice (n=3 each). NS, not significant.

We finally tested whether K114 inhibits the growth of cancer cells. Our unpublished survey revealed that the UPR is constitutively activated in human breast cancer-derived cell line T47D, as evidenced by constitutively elevated levels of *BiP* mRNA (target of the ATF6 pathway), spliced *XBP1* mRNA, *ERdj4* mRNA and *EDEM1* mRNA (targets of the IRE1 pathway), and *CHOP* mRNA (target of the PERK pathway), compared with HCT116 cells (Fig. 4A). Accordingly, 4μ8C inhibited the growth of T47D cells more effectively than that of HCT116 cells at pH 7.2 after 3 days incubation (Fig. 4B); it was previously reported that 4μ8C was effective at a range of 10 μM in a cell culture system (Cross *et al.*, 2012). Moreover, because the tumor microenvironment is acidic and such acidosis is shown to activate the UPR (Dong *et al.*, 2017), 10 μM 4μ8C markedly inhibited the growth of both HCT116 and T47D cells at pH 6.0 (Fig. 4C). Similarly, K114 exhibited the tendency to inhibit the growth of T47D cells more effectively than that of HCT116 cells at pH 7.2 (Fig. 4B), and 10 μM K114 potently inhibited the growth of T47D cells at pH 6.0 (Fig. 4C). Nonetheless, because we cannot rule out the possibility that K114 is cytotoxic to cells, K114 is not considered to be a lead compound and will not be subjected to further analysis.

Instead, we claim that this is the first development of an *in vivo* assay method for IRE1α inhibitors. It is simple and rapid, requiring only embryos, 96-well plates and a microscope. This phenotypic assay can be used as a quick test for the efficacy of IRE1α inhibitors before examining their effects in mice, which is much more laborious process.

## Abbreviations

DKO: double knockout
dpf: day post-fertilization
ER: endoplasmic reticulum
KO: knockout
UPR: unfolded protein response
